# Ingredients for Responsible Machine Learning: A Commented Review of *The Hitchhiker’s Guide to Responsible Machine Learning*

**DOI:** 10.1007/s44199-022-00048-y

**Published:** 2022-09-15

**Authors:** Fernando Marmolejo-Ramos, Raydonal Ospina, Enrique García-Ceja, Juan C. Correa

**Affiliations:** 1 Centre for Change and Complexity in Learning, University of South Australia, Adelaide, SA 5001 Australia; 2 CASTLab, Department of Statistics, Universidade Federal de Pernambuco, Recife, Pernambuco 51280-000 Brazil; 3 Escuela de Ingeniería y Ciencias, Tecnológico de Monterrey, 64849 Monterrey, Nuevo León Mexico; 4 CESA Business School, Bogotá, Bogotá, DC, 110231 Colombia

**Keywords:** Machine learning, Predictive statistics, Inference, Causality

## Abstract

In *The hitchhiker’s guide to responsible machine learning*, Biecek, Kozak, and Zawada (here BKZ) provide an illustrated and engaging step-by-step guide on how to perform a machine learning (ML) analysis such that the algorithms, the software, and the entire process is interpretable and transparent for both the data scientist and the end user. This review summarises BKZ’s book and elaborates on three elements key to ML analyses: inductive inference, causality, and interpretability.

## Introduction

Complex, varied, and big data sets are being amassed rapidly in different fields thanks to digitisation. In the field of health sciences, for example, such data sets have been emerging due to the COVID-19 pandemic [77]. Making sense of such types of data requires powerful and sophisticated computational, mathematical, and statistical tools. Machine learning (ML) is a favourite approach to deal with those data sets as it consists of computer algorithms tuned to automatically find patterns in data [33]. One of the major criticisms of ML, though, is that the algorithms’ internal workings are not tailored to human understanding. Biecek et al. [11] provide a concise, accessible, and engaging tutorial on how to carry out ML analyses that use powerful algorithms in a way that allows both the data scientist and the end user to interpret the workings of the ML analytical process (see also Murdoch et al. [63]). Following canonical book reviews, we summarise and briefly comment on BKZ’s book. BKZ’s book is rich in concepts relating to statistical learning, statistical modelling, and computational statistics, that could be further commented on. However, we chose to elaborate on three concepts that the reader should keep in mind while reading BKZ’s book because they are key to ML and any other form of data analysis: inductive inference, causality, and interpretability.

## A Commented Summary of the Book

BKZ’s book presents the way three fictional data scientists—Bit, Beta, and DALEX—undertake an ML analysis of a COVID-19 data set. While Bit is eager to have quick results, Beta is more cautious and diligent in undertaking further checks and inspecting more predictive models. DALEX is a robot (akin to a friendly version of a Dalek!) that demands explanations of the models built and prompts Bit and Beta to provide these at key steps during the model building. The conceptual foundations underlying these data scientists’ analytical pipeline are grounded in proposals found in Breiman [14] and James et al. [46]. Those conceptual foundations are further developed in more detail in a book by one of the authors Biecek et al. [10].

Bit and Beta are tasked to come up with a predictive model able to determine the risk of death in case of an infection and suggest the age order in which people need to be vaccinated. That is, the data scientists have to sort patients by their individual risks. Bit and Beta thus commence reading up on the topic of COVID-19 to familiarise themselves with the terminology and related aspects. Also, as no data are given to them, they start to find a comparable and representative data set with which to build the predictive model. This step is crucial in that the data set Bit and Beta use will be the data generating process (DGP) substantiating any statistical model such that any subsequent explanation and prediction is directly dependent on the DGP.

With the data at hand, Bit and Beta create a training data set and a test data set (in BKZ’s book, these are the COVID_spring and COVID_summer data sets, respectively). The former is used to build the model and the latter is used to validate the model. BKZ’s book briefly comments on a key aspect; a true validation is done on a separate new data set. Note that ‘true validation’ is different from cross-validation (sometimes called rotation estimation or out-of-sample testing). Different from a true validation approach, in cross-validation the original data set is split in such a way that a large chunk of the data (say, 80%) is used to train the model, and the remaining data is used to test the model [82]. Cross-validation, or any form of model assessment, is at the core of model building in that it enables examination of the stability of the model’s estimations [92]. Anecdotally, cross-validation predates bootstrapping [25], an influential technique used in statistical modelling [34], and these two techniques can be used in conjunction in ML analyses [84].

Bit and Beta move onto exploring (via exploratory data analysis [EDA] techniques [85]) and cleansing the data. When these steps are cleared, they are ready to consider statistical algorithms suitable to the data and the research problem at hand. This is the stage where the predictive power of some (binomial) classification algorithms is assessed via DALEX. At this point the reader realises that DALEX is a robot that embodies the DALEX R package, a package designed for assessing and explaining predictive models [9]. Bit, Beta, and DALEX first try a regression tree that uses the variables ‘age’ and ‘cardiovascular diseases’ (that these variables were used by the regression tree is not surprising as these variables were also highlighted during the EDA phase of the data analysis). The data scientists then try an algorithm that is an improvement on regression trees: random forests. The results are better and after some optimisation of the hyperparameters (i.e. tuning) the diagnostic ability of the binary classifier improves even more. BKZ explain how to optimise hyperparameters and evaluate the importance of the data set’s variables. The examination of the variables is furthered via partial dependence (PD) and accumulated local effects (ALE). We will not expand on hyperparameter optimisation, variable importance, PD, and ALE as BKZ already do this in their book. Regarding classification algorithms, it is important to note that although classification trees and random forests provide good visuals of decision trees, there are other algorithms that can assist in classification tasks. There are, for example, the one rule (1R) [42] and the Boruta [52] algorithms. A logistic regression algorithm could also be considered as it has been shown this method is more interpretable than, yet similarly accurate to, more complex ML algorithms [19, 54, 57, 64]. Note that it is indeed possible to combine classification algorithms in order to inform a final model. For example, Cardona et al. [16] used the Boruta and 1R algorithms for selecting variables to be used in a logistic regression model. In the case of numeric dependent variables, techniques such as distributional regression trees and forests [74] and transformation forests [44] could be used (these are implemented in the disttree and trtf R packages, respectively).

Bit, Beta, and DALEX inspect their models further through Shapley values (a concept from cooperative game theory), break-down plots, and ceteris paribus plots (a.k.a. what-if plots). Once again, these concepts are clearly explained in BKZ’s book but other sources such as Biecek and Burzykowski Molnar [10] and [60] are recommended. Once the three data scientists are satisfied with the results of the further assessment of the models and the results of some individual risk analyses, they are thus finally ready to deploy the model. The three data scientists create an application that allows any individual to estimate the probability of severe condition and death after being diagnosed with COVID-19 depending on age, gender (male or female), presence/absence of cardiovascular disease, presence/absence of cancer, presence/absence of kidney disease, presence/absence of diabetes, and presence/absence of other diseases (the app lives at https://crs19.pl/). In the app’s page, it is made explicit that the model is built using a sample of 50,000+ cases in Poland who gave a positive PCR (polymerase chain reaction) test for COVID-19. Other important information about the data set, variables, and models is provided therein.

In a nutshell, BKZ’s book argues that responsible ML consists of preparing the data, understanding it, proposing an ensemble of models to parse the data (based on the research question), carefully auditing the models, and finally deploying the models. Thus, BKZ’s book sets an example of what good practices and principles in explainable ML should look like [6]. As mentioned earlier, BKZ’s book is rich in concepts that cut across, mostly, the fields of statistical and computer sciences. We chose three concepts central to data science in general (including ML) and we consider them in turn.

## Inductive Inference

Inductive inference [2, 3, 22] can be understood as an ML procedure [7, 12, 70, 78] or algorithm [29, 37] that assumes a specific type of relationship between hypotheses about the data and propositions that go beyond the data (and these include predictions about future data, general conclusions about all possible data, and the DGP) [20].

Inductive inference aims to provide the best predictions and identify the best model for inferential purposes (variable selection, hypothesis testing, etc.) that allow the generation of scientific knowledge and interpretation. A key premise, though, is that simple models are preferable [18, 94]. Inductive inference requires assumptions for the application of statistical tests; however, from an ML perspective, an algorithm, by definition, is a set of finite steps that become an inductive inferential process in itself [79, 80, 91]. That is, any assumption check built into statistical testing is stripped by inferential processes carried out by algorithms [36, 69].

The language used to describe patterns in the data, sample size [53], computational complexity of problems [67] in approximating concepts [30, 72], and poor pattern identification methods further adds a layer of complexity to inductive inference. The way those domains are described can induce biases in inductive inferential reasoning [47] (an example of this can be found in several probabilistic problems) [5, 15, 48, 50, 71, 83].

In the specific case of interpretation of results obtained via ML, it has been argued that ML researchers tend to incur the illusion of probabilistic proof by contradiction, which consists of the erroneous belief that a null hypothesis becomes improbable because a significant result has been obtained [27, 28]. This illusion is, however, difficult to eradicate in the use of inductive inference. Given that BKZ’s book embraces an ML approach, it does not stress the importance of the verification of hypotheses, attention to the limits of extrapolating results [40, 45, 81], and securing corrective measures [26, 59]. We strongly believe that these are aspects in inductive inference to which future work in ML should give serious consideration.

## Causality

In statistics and ML literature, causality or causal inference (i.e., deciding whether a variable X causes Y or vice versa) is one of the most debated topics in the academic community. The possibility of making causal inferences represents an ideal mechanism for any scientist trying to uncover natural laws, and traditional approaches to uncovering these laws favour controlled experiments [38, 41, 62]. Besides controlled experiments, more recent data-driven perspectives suggest other techniques for causal inference purposes in experimental and non-experimental contexts [13, 75]. BKZ’s book’s position regarding this topic is evident: predictive models are mentioned without implying any connection to causality or causal inference. Such a pedagogical position, we believe, not only mirrors the infancy that describes the current stage of the literature on ML and causality, but also exploits the data of COVID-19 to illustrate how different ML models can be used in R and how they provide several approaches to the same problem: modelling individual mortality risk after COVID-19 infection.

In our view, even though the topic of causality was not covered in BKZ’s book, the reader is encouraged to understand that this topic cannot be ignored. Regardless of existent contrasting views on the possible ways to make causal inferences out of ML models, there will always be relevant spaces for discussing these classic concerns in statistical reasoning. For example, Bontempi and Flauder [13] proposed a supervised ML approach to infer the existence of a directed causal link between two variables in multivariate settings with $n > 2$ variables. By the same token, the idea of discriminating cause from effect with observational non-experimental data is well introduced by Mooij et al. [62]. Since then, another branch of the literature presents interesting insights about the way researchers can learn causality from data [38, 65, 93]. In line with the working paper of Schölkopf [75], we also believe that the hard open problems of ML and AI are intrinsically related to causality and that this is another central topic requiring more attention from ML researchers.

## Interpretability (explainable ML and AI)

Pedagogical efforts like the one provided by BKZ are undoubtedly helpful in an era where several institutions leverage ‘black-box’ ML models for high-stakes decisions (e.g. healthcare and criminal justice) [73]. The utility of these efforts is evident when it comes to illustrating how ML models work in general and how they reach their predictions in particular. In our view, the use of COVID-19 data makes BKZ’s book a clear and updated reference and highlights their unique intended goal: finding a balance between technicalities and possible pedagogical illustrations through funny adventures of comic characters. In just 54 pages, the book does not pretend to dive deep into the inner workings of the methods. Nonetheless, it provides a good sample of appropriate references and serves as an intuitive starting point for beginners. A more expert audience might find helpful other sources that invest more pages for similar purposes without the pedagogical resource of comics [49].

BKZ make the distinction between two types of ML interpretable methods: global model-based and instance-based. Examples of both types of methods are presented. One thing to note is that the primary focus of the book is on explainable methods for *supervised* ML and *tabular data*. Given recent advances in algorithms that work on more unstructured data such as text, images, and time series of varying length, explainable ML methods have also permeated into those domains. For example, Assaf and Schumann [4] proposed a deep neural network to explain time series predictions. Liu et al. [55] developed a framework for generating explanations for natural language processing tasks; specifically, text classification. Furthermore, in recent years explainable AI methods outside the supervised learning domain have been developed, for example, for unsupervised clustering [23, 32] and reinforcement learning [68] (see also Bhatti [8]).

One aspect that is closely related to explainable ML and that is not covered in BKZ’s book (and is also left aside in many other explainable ML materials) is a model’s *uncertainty quantification*. By design, many ML models always produce a prediction regardless of their quality and without providing guarantees of their uncertainty; for example, *p*-values for classification and confidence intervals for regression. In medical applications and other domains it is of critical importance to know if a model’s prediction can be trusted; alas, such information is not usually available. Many models like neural networks, decision trees, K-NN, and so on can produce prediction scores or probabilities; however, those are relative to the given data point and class (in the case of classification) but do not necessarily represent the overall probability distribution. When analysing predictions, it is important to consider both their explanations and their trustworthiness. The later can be assessed with *conformal prediction*, which is a framework proposed by Vovk et al. [90] to estimate the predictions’ uncertainty. One of the advantages of this framework is that it is model agnostic. A recent method for uncertainty estimation was also proposed by Sensoy et al. [76]; however, it is specific to deep learning models.

There are several implementations of many explainable ML methods in the form of R packages. An extensive list of 27 packages was compiled and analysed by Maksymiuk et al. [58], with DALEX [9], lime [66], and iml [61] being some of the most popular (based on GitHub stars). Some R packages for general ML are implemented in EnsembleML, cvms (cross-validation for model selection), and MachineShop (see also the CRAN site on ML at https://cran.r-project.org/web/views/MachineLearning.html). Finally, there is another ML-related technique that the reader of BKZ’s book should be aware of that is known as ‘targeted learning’. This approach relies on ML algorithms to assess uncertainty and provide reliable estimations of the true target parameters of the probability distribution of the data [86–89] (an online free book can be found at https://tlverse.org/tlverse-handbook/ and the key R packages are SuperLearner and tmle). In our view, BKZ’s book invites the reader to conceive ML analyses and models that are interpretable so that their utility is optimised.

## Final Thoughts

ML is a technique that automates data analysis by resorting to the power of statistical tools [21, 31] and has become a favoured framework to cope with big data by producing predictive models [24] across several fields [1, 43, 51, 56]. However, those models are known for lacking interpretability and explainability [17] and this, in turn, reduces their accountability because issues relating to risk assessment and safe adoption are overlooked [39]. BKZ’s book aims to alleviate that problem by providing a concise and engaging tutorial on how to carry out careful and responsible ML analyses. We thus recommend their book as complementary reading for those undertaking ML-related courses. Different from current introductory textbooks on ML (e.g. Ghatak [35]), BKZ’s book shows that an ML-based analysis is not about fiddling with black-box algorithms and praying for the best. Instead, the authors show that ML-based analyses require carefully selecting and tuning algorithms that, while giving accurate predictions, retain a good level of interpretability and explainability. That is, the ML analysis and analyst become responsible. We believe this message applies not only to ML modelling but to all forms of data analysis.

## Data Availability

No datasets were generated or analysed during the current study

## Abbreviations

ML: Machine learning
BKZ: Biecek, Kozak, and Zawada
COVID-19: Coronavirus disease 2019
DGP: Data generating process
EDA: Exploratory data analysis
PD: Partial dependence
ALE: Accumulated local effects
1R: One rule algorithm
PCR: Polymerase chain reaction
K-NN: k-Nearest neighbors algorithm
